# The Role of IFN‐γ‐Mediated Immune Cell Crosstalk in the Pathogenesis of Aplastic Anemia

**DOI:** 10.1155/jimr/2216487

**Published:** 2026-02-27

**Authors:** Shuai Tan, Yumeng Li, Yaochi Chen, Wanling Sun

**Affiliations:** ^1^ Department of Hematology, Xuanwu Hospital, Capital Medical University, Beijing, China, ccmu.edu.cn; ^2^ Comprehensive Center for Neuro-Oncology, Xuanwu Hospital, Capital Medical University, Beijing, China, ccmu.edu.cn; ^3^ National Center for Neurological Disorders, Beijing, China, fudan.edu.cn

**Keywords:** aplastic anemia (AA), crosstalk, IFN-γ, macrophage, platelet, T cell

## Abstract

Interferon‐gamma (IFN‐γ) is a central mediator of immune‐driven bone marrow failure (BMF) in acquired aplastic anemia (AA). Persistent IFN‐γ signaling alters the bone marrow microenvironment by activating the JAK–STAT1 pathway, which results in immunological imbalance, inflammatory amplification, and depletion of hematopoietic stem and progenitor cells (HSPCs). IFN‐γ disturbs HSPC quiescence and self‐renewal, interferes with thrombopoietin (TPO)–c‐Mpl communication, and stimulates cytotoxic T‐cell‐dominant immunological responses. Simultaneously, IFN‐γ destabilizes local immunological homeostasis by disrupting immune crosstalk through the IDO1 axis and regulatory T‐cell (Treg) malfunction. In addition to discussing new therapeutic methods, such as Treg‐based therapies and JAK inhibition, as prospective precision approaches for AA, this review incorporates current mechanistic insights into IFN‐γ‐driven cellular interactions inside the bone marrow niche.

**Summary**

• Explains in detail how IFN‐γ uses the JAK–STAT1 pathway to modify the bone marrow microenvironment.
• Explains how Interferon‐gamma (IFN‐γ) causes hematopoietic stem and progenitor cell (HSPC) fatigue by interfering with thrombopoietin (TPO) receptor signaling.
• Examines Treg impairment and immunological communication dysregulation caused by IFN‐γ via the IDO1 axis.
• Assesses Treg‐based treatments and JAK inhibitors as new precision therapeutic approaches for aplastic anemia (AA).

## 1. Introduction

**Figure 1:**
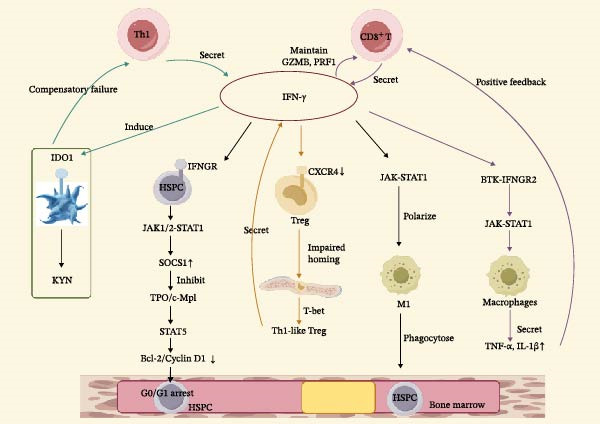
IFN‐γ–Driven Immune Dysregulation in Aplastic Anemia. This figure outlines an IFN‐γ–centered immune network in aplastic anemia. IFN‐γ produced by Th1 and CD8^+^ T cells activates JAK–STAT1 signaling, suppresses HSPC maintenance, disrupts Treg homeostasis, and promotes pro‐inflammatory macrophage polarization, collectively driving bone marrow microenvironment dysfunction and hematopoietic failure. (This figure was made by “figdraw.com”).

Acquired aplastic anemia (AA) is an immune‐mediated bone marrow failure (BMF) disorder, characterized by pancytopenia and bone marrow hypoplasia [1]. The classical pathogenic model emphasizes hematopoietic stem and progenitor cell (HSPC) damage mediated by aberrant T cell activation, with type 1 T helper (Th1) polarization and the resulting Interferon‐gamma (IFN‐γ) surge recognized as central contributors to hematopoietic failure [2, 3]. Historically, the prognosis of severe AA (SAA) was poor. The 2‐year mortality rate for patients with SAA or very SAA (VSAA) who receive just supportive therapy is approaching 80% [4]. Current treatment options, including hematopoietic stem cell transplantation (HSCT) and immunosuppressive therapy (IST), have improved survival rates to 80%–85% [5], however, the response rate to standard IST remains only 50%–70%, indicating that, beyond T cell dysfunction, other critical pathogenic mechanisms may be involved [6, 7]. IFN‐γ plays a pivotal role in the pathogenesis of AA and is a key regulator of HSPC growth inhibition and apoptosis in BMF syndromes [8]. Recent studies have revealed two additional contributing factors: (1) nonlymphoid cells within the bone marrow microenvironment, such as macrophages and platelets, can exacerbate inflammation by secreting proinflammatory cytokines [7, 9]; and (2) the cytokine network involves multiple cellular sources and displays bidirectional regulatory functions, intensifying BMF under inflammatory conditions [10]. This dysregulation has not been comprehensively examined. IFN‐γ is regarded as a core mediator of the “cytokine storm,” driving BMF in AA through cascade amplification of inflammatory responses [8]. Therapeutically targeting and inhibiting its downstream signaling pathways, such as with JAK1/2 inhibitors, may disrupt this vicious cycle and offer a novel treatment strategy for AA (Figure 1).

## 2. IFN‐γ and Its Receptor

IFN‐γ is a type II interferon secreted by activated T cells, particularly cytotoxic T lymphocytes (CTLs), Th1 cells, type 1 innate lymphoid cells (ILC1s), and macrophages [11]. Its function depends on a receptor complex composed of interferon gamma receptors (IFNGR1 and IFNGR2), located on the surface of various nucleated cells. IFNGR1 directly binds to IFN‐γ, while IFNGR2 dimerizes with IFNGR1 to facilitate downstream signal transduction. Loss of IFNGR1 completely abolishes signal transduction, whereas loss of IFNGR2 leads to partial attenuation of signaling [12]. Protein cross‐linking experiments have shown that IFNGR1 can dimerize independently and internalize, and that IFN‐γ binds to IFNGR2 only through its association with IFNGR1 [13], confirming the central role of IFNGR1 in receptor activation. IFN‐γ signaling is primarily mediated through the JAK‐STAT pathway [14]. JAK1/JAK2 phosphorylates STAT1 upon receptor contact, resulting in the formation of a phosphorylated homodimer. This dimer exerts regulatory and effector effects by translocating into the nucleus, binding to the IFN‐γ activated sequence (GAS), and directly driving the transcription of IFN‐γ‐stimulated genes (ISGs) [12, 15].

## 3. The Fundamental IFN‐γ Signaling Pathway Provides a Molecular Basis for Immune Regulation in AA

Within the bone marrow microenvironment of AA, the canonical JAK–STAT1 pathway functions not merely as a downstream effector pathway but as a “molecular engine” that drives pathological intercellular communication [16, 17]. By inducing a broad spectrum of ISGs, IFN‐γ signaling reshapes the phenotypes of T cells, macrophages, and platelets in the bone marrow. It reinforces Th1 polarization through the STAT1–T‐bet axis [18] and sustains the inflammatory milieu by modulating macrophage responsiveness [19]. This dynamic flow of molecular signaling transforms otherwise isolated cellular responses into an interconnected pathogenic pathway, collectively establishing a hostile microenvironment that targets HSPC.

### 3.1. IFN‐γ Mediated Communication of Immune Cells and the Mechanism of AA

#### 3.1.1. IFN‐γ and Macrophages

IFN‐γ is a key inducer of M1 macrophage polarization [15]. In an E‐selectin‐deficient mouse model, inflammation‐induced E‐selectin was shown to activate Bruton’s tyrosine kinase (BTK), which phosphorylates cytoplasmic IFNGR2 at Tyr289. This facilitates EFhd2 binding, promotes IFNGR2 translocation from the Golgi to the plasma membrane, and enables assembly of a functional IFNGR1/2 complex, significantly enhancing macrophage sensitivity to IFN‐γ [12];.

Upon receptor binding, IFN‐γ exerts its effects primarily by activating the JAK–STAT1 pathway. STAT1 cooperates with the lineage‐determining factor PU.1 to form “primed enhancers” facilitating chromatin opening. Simultaneously, it suppresses anti‐inflammatory genes such as PPARG and MERTK via histone H3 lysine 27 trimethylation (H3K27me3) and inhibits activation of anti‐inflammatory genes in M2 macrophages by blocking H3K27 acetylation (H3K27ac) signaling [20–22]. IFN‐γ impairs mitochondrial function by inhibiting the mTORC1 pathway, thereby enhancing proinflammatory cytokine expression [23]. Under conditions of energy stress, it activates AMPK, suppresses mTORC1, and helps sustain the M1 phenotype [24]. IFN‐γ also activates glycogen synthase kinase 3 (GSK3), which counteracts the inhibitory effect of cAMP response element‐binding protein (CREB) on NF‐κB, further promoting transcription of proinflammatory genes [25]. Interferon regulatory factor 1 (IRF1) plays a crucial role in the delayed‐phase response [26], working synergistically with LPS–TLR4‐activated NF‐κB and IRF3 to form the ISGF3 complex, which continuously amplifies proinflammatory signaling via interferon‐stimulated response elements (ISREs) [27].

In AA immunopathogenesis, IFN‐γ functions as a “signal flag” recruiting bone marrow macrophages. In SAA patients, elevated IFN‐γ activates the JAK–STAT1 pathway (Kawakami et al. [28] showed that bicarbonate can synergize with IFN‐γ to amplify this signaling), driving macrophages toward M1 polarization. M1 macrophages then excessively phagocytose residual HSPC, directly impairing hematopoiesis. This creates a vicious feedback loop. In AA mouse models, M1 macrophage depletion lowers bone marrow TNF‐α and suppresses CTL responses, reducing IFN‐γ production [7]. These findings highlight M1 macrophages as central mediators of the immune cascade driving BMF.

### 3.2. IFN‐γ and T Cells

In the AA microenvironment, IFN‐γ hypersecreted by Th1 and CD8^+^T cells is the primary driver of hematopoietic suppression. Binding to IFNGR1/2 on HSPC, it activates the JAK1/2‐STAT1 pathway to initiate an “interferon‐response program” [29]. This axis rapidly upregulates the negative regulator suppressor of cytokine signaling 1 (SOCS1) and inhibits STAT5 phosphorylation downstream of survival receptors such as thrombopoietin (TPO) and its receptor c‐Mpl (TPO‐c‐Mpl), thereby blocking the Bcl‐2/Cyclin D1 signaling pathway. This forces HSPCs to arrest at the G0→G1 transition phase, preventing them from completing self‐renewal [30]. Chronic exposure to IFN‐γ leads to impaired self‐renewal capacity and enhanced myeloid differentiation bias of HSC, ultimately resulting in HSC pool exhaustion [31].

In addition, Th1 cells can promote CD8^+^ T cell activation to further secrete inflammatory cytokines such as IFN‐γ, accelerating disease progression [32]. Sustained elevated IFN‐γ upregulates granzyme B and perforin in CD8^+^ T cells via JAK–STAT1, maintaining their cytotoxic function and amplifying hematopoietic suppression. This forms a malignant positive feedback loop of “T cells‐IFN‐γ‐hematopoietic suppression," which serves as the core axis of immune attack in AA [33]. Research has demonstrated that SAA patients with gain‐of‐function (GOF) mutations in STAT1 had activated cytotoxic memory CD8^+^ T cells and elevated plasma IFN‐γ levels without previous IST. This implies that aberrant amplification of IFN‐γ signaling due to enhanced STAT1 function reshapes CD8^+^ T cell phenotype and activates IFN‐γ‐responsive myeloid cells. It thereby initiates a sustained inflammatory positive feedback pathway centered on IFN‐γ–STAT1 and sustained by chemokines such as CXCL10 in the bone marrow, which continuously increases T cell‐mediated HSPC destruction and propels eventual hematological failure [17].

Through the IL‐2/Treg/IFN‐γ negative feedback loop, Tregs defend HSCs: IL‐2‐sustained Tregs prevent effector T cells in the bone marrow from producing pathogenic IFN‐γ. When this equilibrium is upset, there is an increase in IFN‐γ, which disrupts HSC Notch‐Runx signaling, reduces quiescence, and results in hematopoietic failure [34, 35]. Numerous investigations have shown that the pathophysiology and development of AA are significantly influenced by decreased Treg frequency and numbers as well as functional abnormalities in the bone marrow and peripheral circulation [36, 37]. Due to downregulated CXCR4, Tregs in AA have poor bone marrow homing in both peripheral blood and bone marrow, which lessens their ability to inhibit effector T cells locally. Functionally, Tregs generated from AA patients exhibit a significantly diminished ability to regulate Th1/CTL proliferation and IFN‐γ production, resulting in persistently elevated IFN‐γ levels that worsen hematopoietic suppression and increase effector T cell activation [38]. Excessive IFN‐γ disrupts T‐cell homeostasis by reprogramming Tregs into T‐bet+Th1‐like cells [39]; in models of BMF, unrestrained IFN‐γ secretion coincides with Treg depletion and HSC apoptosis [40].

### 3.3. IFN‐γ and Platelets

In a murine malaria model, IFN‐γ induces the expression of indoleamine 2,3‐dioxygenase 1 (IDO1) in platelets through extrinsic signaling, leading to an increased plasma kynurenine/tryptophan (KYN/TRP) ratio, enhanced Trp catabolism toward the KYN pathway, and consequent suppression of CD4+/CD8+ effector T‐cell functions [41]. Although TRP catabolism is acknowledged in conventional theory as an immunosuppressive feedback loop that limits excessive T cell activation, in the highly inflammatory microenvironment of AA, persistent Th1‐driven signals frequently overwhelm this compensatory axis [42, 43]. Platelet‐derived platelet factor 4 (PF4) drives atherosclerotic progression through distinct yet interconnected pathways. By activating the CXCR3‐Akt‐PGC1α‐TFAM axis, PF4 enhances mitochondrial biogenesis in Th1 cells, leading to increased IFN‐γ secretion that promotes endothelial inflammation and macrophage‐derived foam cell formation. Although PF4 also activates FOXP3+Treg cells to secrete the anti‐inflammatory cytokine IL‐10, their immunosuppressive function is counteracted by enhanced Th1 activity, leading to uncontrolled inflammation [44, 45].

## 4. IFN‐γ and AA

AA is characterized by BMF and peripheral pancytopenia, closely associated with elevated levels of IFN‐γ [46]. Plasma IFN‐γ levels in AA patients are considerably greater than those in healthy controls, according to a peripheral blood cytokine profiling study. This is frequently accompanied by concurrent elevation of Th1‐type inflammatory cytokines such as TNF‐α and IL‐2. This suggests that the immune abnormalities in AA are not limited to the bone marrow but have a systemic immunological basis, which can provide contextual support for sustained T cell activation and hematopoietic suppression. This suggests that AA is in a state of systemic immune activation characterized by Th1 immune bias [47]. Sustained low‐level IFN‐γ expression can directly affect HSPCs in IFN‐γ adenylate‐uridylate‐rich element (ARE)‐deleted (del) mice models. It modifies HSPC composition, disrupts lineage differentiation, and eventually causes AA‐like symptoms in the absence of bone marrow T cell infiltration [48]. IFN‐γ simultaneously activates the STAT3 signaling pathway and strengthens Th1 polarization through the STAT1/T‐bet positive feedback loop. This process creates an IFN‐γ/STAT1–STAT3 balancing regulatory node with STAT1 in addition to impairing the quiescence of HSC, which results in their excessive proliferation and exhaustion. Hematopoietic suppression is further intensified by signal imbalance via this node [49–52].

A combined hematopoietic suppression mechanism known as “receptor blockade and signal interference” can be formed by IFN‐γ directly binding to the TPO receptor c‐MPL to block its activity and activating STAT1/STAT3 signaling to disrupt the c‐MPL‐JAK2/STAT5 hematopoietic support axis [53, 54]. Furthermore, IFN‐γ causes immune cells (such as platelets and macrophages) to express IDO1, which starts the metabolic reprogramming of the IDO1–KYN axis. It suppresses the immunomodulatory activity of Tregs and indirectly increases the pathogenicity of Th1/CD8^+^ T cells by depleting Trp and generating KYN [41, 44]. In conclusion, IFN‐γ and its downstream cross‐signaling pathways are key therapeutic targets for AA because cross‐signaling amplification among these important nodes produces a synergistic effect that collectively exacerbates hematopoietic stem and HSPC damage and hematopoietic failure [55] (As shown in Figure 1).

## 5. Summary and Future Perspectives

New treatment options for AA have been made possible by research focusing on IFN‐γ and its downstream signaling pathways; however, there are still significant obstacles in getting these discoveries from the bench to the patient’s bedside. In AA patients with GOF mutations in STAT1, JAK medications, including itacitinib, have shown initial clinical benefit [17]. These medications successfully raise peripheral blood counts and aid in the reprogramming of the dysregulated immunological microenvironment by blocking STAT1 phosphorylation. Nonetheless, prolonged use of nonselective JAK/STAT inhibitors may induce off‐target effects, interfering with normal hematopoietic cytokine signaling—specifically TPO pathways—resulting in secondary suppression of hematopoiesis [56]. Furthermore, even though neutralizing IFN‐γ is considered a crucial tactic for reversing hematopoietic suppression, its clinical implementation necessitates rigorous risk assessment. If IFN‐γ is directly blocked, antiviral and antitumor immune surveillance may be compromised, making people more vulnerable to serious infections [57]. Future research should concentrate on the exact manipulation of crucial downstream nodes in order to address issues with toxicity and specificity. For instance, blocking IFNGR2 membrane trafficking in macrophages by targeting the E‐selectin/BTK/EFhd2 axis may allow local IFN‐γ‐driven inflammatory cascades to be blocked without changing systemic interferon levels. Furthermore, strategies that combine low‐dose IL‐2 administration with Treg adoptive transfer to reconstitute the IL‐2/Treg/IFN‐γ negative feedback regulatory loop may be more biologically rational than nonspecific immune depletion alone, given the decreased frequency and functional impairment of Tregs in AA patients [34].

In the future, multi‐omics‐guided tailored medicine should replace empirical immunosuppression in the treatment of AA. It may be able to pinpoint the fundamental molecular switches causing BMF by dynamically tracking cytokine kinetics and receptor expression patterns at various phases of the disease’s course. In addition to providing precision therapy choices for refractory cases that are not responding to traditional medications, such a strategy would enable focused therapeutic approaches that reduce toxicity.

## Author Contributions

Shuai Tan and Yumeng Li performed the study. Shuai Tan, Yumeng Li, Yaochi Chen, and Wanling Sun performed literature review, interpreted the data, and wrote the manuscript. Shuai Tan and Yumeng Li interpreted the data, wrote the manuscript, and revised the manuscript. Wanling Sun designed the study, interpreted the data, and organized the research.

## Funding

This study was supported by the National Outstanding Youth Science Fund Project of National Natural Science Foundation of China (Grant 82300161), the Beijing High Innovation Program·Spring Bud Project (Grant 2‐1‐008‐0262), the Scholar Support Program of Huizhi Talent Project of Xuanwu Hospital, Capital Medical University (Grant HZ2025ZCYX003);Beijing Future Talent Training Program in Medical‐Engineering Field (Grant MBRC0012025063); the Natural Science Foundation of Beijing Municipality (Grant Z200022); the Beijing High‐level Overseas Returnee Talent Funding Project (Grant 2‐2‐008‐0243), the Capital Medical University Science and Innovation Elite Plan Project (Grant 2024KCJY0405), the “National Natural Science Foundation of Youth Cultivation Project” of “Xuanwu Hospital, Capital Medical University, Beijing, China,” (Grant QNPY2022014), the “Person of Outstanding Ability Training Program” of “Xuanwu Hospital, Capital Medical University, Beijing, China” (Grant YC20220127).

## Disclosure

All authors provided critical feedback during manuscript preparation and approved the final version for publication.

## Conflicts of Interest

The authors declare no conflicts of interest.

## Data Availability

Data sharing is not applicable to this article, as no datasets were generated or analyzed during the current study.
